# New Inventions

**Published:** 1895-08

**Authors:** 


### NEW INVENTIONS.

A currycomb, with a brace of V-shaped plates, with a series of vertical slats, forming a series of rounded teeth.

A horseshoeing machine, also intended for confining animals while performing operations, comprising upright supports, with hinged movable sides, to which a series of girths are attached; an overhead tackle by which the animal may be lifted from the ground; also a windlass at the rear for controlling the hind legs.

A halter with a brow-band, a nose-band, a mouth-piece with halter-ring attachment, and a rear mouth-piece.

A rolled-metal horseshoe-bar with nail-crease, the bevelled side having short teeth or spikes rolled upon it.

A moulded hollow aluminum horse-collar, arranged to be covered with leather and drilled to receive mountings.

A fastening device for horseshoes, an offset on the shoe which is fastened to a hole in the wall of the hoof, arranged by a key.

Drainage system for stables, consisting of a sealed floor with liquid-proof material, sloped toward the centre from the sides, conforming to the contour of the body when lying down, the surface of the floor grooved to facilitate drainage.

A horse-collar with an inflated pneumatic tube covered by metallic strips with valve attachment.

Combination tool for horsemen, arranged in a handle as a pocket-knife, containing a pair of pincers, a claw-hook, or foot-cleaner, a punch, a screwdriver, and several blades.

A stock watering-trough with a water inlet opening and outlet, regulated by a valve-plate attachment.

A hitching-strap, particularly adapted for saddle-horses employed for military purposes, consisting of a strap, a sliding snap-hook, a buckle-frame secured at one end and a buckle secured firmly near the opposite end of the strap, which is converted into a loop, so that the buckle-frame may be engaged or a halter-ring.

A spring horseshoe, consisting of an elastic bridge-piece at toe, uniting the two wings, the bridge bent up and rearwardly at an angle to the wings, having its lower edge above the same.

A horse-collar composed of two semicircular metallic bodies turned inwardly to form tubular rims and made extensible telescopically by thumb-screws. The part bearing on the neck formed of inflated rubber, making air-cushions.

